# Long-term outcome of the Emergency Response Team system in in-hospital cardiac arrest

**DOI:** 10.1186/cc13273

**Published:** 2014-03-17

**Authors:** J Suriyachaisawat, E Surakarn

**Affiliations:** 1 Bangkok Hospital Group, Bangkok, Thailand

## Introduction

To improve early detection and the mortality rate of inhospital cardiac arrest, the Emergency Response Team (ERT) system was planned and implemented since June 2009 to detect pre-arrest conditions and for any concerns. The ERT consisted of on-duty physicians and nurses from emergency department. ERT calling criteria [1],[2] consisted of acute change of HR <40 or >130 beats per minute, systolic blood pressure <90 mmHg, respiratory rate <8 or >28 breaths per minute, O_2 _saturation <90%, acute change in conscious state, acute chest pain or worried about the patients. From the data on ERT system implementation in our hospital in the early phase (during June 2009 to 2011), there was no statistical significance in difference in in-hospital cardiac arrest incidence and overall hospital mortality rate. Since the introduction of the ERT service in our hospital, we have conducted a continuous educational campaign to improve awareness in an attempt to increase use of the service.

## Methods

To investigate the outcome of the ERT system in in-hospital cardiac arrest and the overall hospital mortality rate, we conducted a prospective, controlled before and after examination of the longterm effect of an ERT system on the incidence of cardiac arrest. We performed chi-square analysis to find statistical significance.

## Results

Of a total 623 eRt cases from June 2009 until December 2012, there were 72 calls in 2009, 196 calls in 2010, 139 calls in 2011 and 245 calls in 2012. The number of ERT calls per 1,000 admissions in year 2009 to 2010 was 7.69, 5.61 in 2011 and 9.38 in 2013. The number of Code Blue calls per 1,000 admissions decreased significantly from 2.28 to 0.99 per 1,000 admissions (*P *< 0.001). The incidence of cardiac arrest decreased progressively from 1.19 to 0.34 per 1,000 admissions and was significant in difference in year 2012 (*P *< 0.001). The overall hospital mortality rate decreased by 8% from 15.43 to 14.43 per 1,000 admissions (*P *= 0.095).

## Conclusion

ERT system implementation was associated with progressive reduction in cardiac arrests over the 3-year period, especially statistically significant in difference in the fourth year after implementation. We also found an inverse association between number of ERT use and the risk of occurrence of cardiac arrests, but we found no difference in overall hospital mortality rate.
